# IL-37 Exerts Anti-Inflammatory Effects in Fetal Membranes of Spontaneous Preterm Birth via the NF-*κ*B and IL-6/STAT3 Signaling Pathway

**DOI:** 10.1155/2020/1069563

**Published:** 2020-07-11

**Authors:** Lulu Wang, Zheng Liu, Dongni Huang, Yuxin Ran, Hanwen Zhang, Jie He, Nanlin Yin, Hongbo Qi

**Affiliations:** ^1^Department of Obstetrics, The First Affiliated Hospital of Chongqing Medical University, Chongqing 400016, China; ^2^Chongqing Key Laboratory of Maternal and Fetal Medicine, Chongqing Medical University, Chongqing 400016, China; ^3^Joint International Research Laboratory of Reproduction and Development of Chinese Ministry of Education, Chongqing Medical University, Chongqing 400016, China; ^4^Center for Reproductive Medicine, The First Affiliated Hospital of Chongqing Medical University, Chongqing 400016, China

## Abstract

Spontaneous preterm birth (sPTB), defined as delivery before 37 weeks of gestation, is thought to be a multifactorial syndrome. However, the inflammatory imbalance at the maternal-fetal interface promotes excessive secretion of inflammatory factors and induces apoptosis and degradation of the extracellular matrix (ECM), which can subsequently lead to preterm birth. As an anti-inflammatory molecule in the IL-1 family, interleukin-37 (IL-37) mainly plays an inhibiting role in a variety of inflammatory diseases. However, as a typical inflammatory disease, no previous studies have been carried out to explore the role of IL-37 in sPTB. In this study, a series of molecular biological experiments were performed in clinical samples and human amniotic epithelial cell line (Wistar Institute Susan Hayflick (WISH)) to investigate the deficiency role of IL-37 and the potential mechanism. Firstly, the results indicated that the expression of IL-37 in human peripheral plasma and fetal membranes was significantly decreased in the sPTB group. Afterward, it is proved that IL-37 could significantly suppress the production of tumor necrosis factor-*α* (TNF-*α*), interleukin-1*β* (IL-1*β*), and interleukin-6 (IL-6) in WISH cells. Simultaneously, once silence IL-37, LPS-induced apoptosis and activity of matrix metalloproteinases (MMPs) 2 and 9 were significantly increased. In addition, the western blot data showed that IL-37 performed its biological effects by inhibiting the NF-*κ*B and IL-6/STAT3 pathway. In conclusion, our results suggest that IL-37 limits excessive inflammation and subsequently inhibits ECM remodeling and apoptosis through the NF-*κ*B and IL-6/STAT3 signaling pathway in the fetal membranes.

## 1. Introduction

Spontaneous preterm birth (sPTB), occurring before 37 weeks of gestation, is a specific disorder of pregnancy. With around 15 million preterm infants born each year, accounting for approximately 10.6% of all live births worldwide, sPTB is considered to be the most frequent cause of mortality in neonatal and the second in children age below 5 years old worldwide [1, 2]. Furthermore, those survived preterm neonates have higher rates of multiple complications, including neurological dysplasia, respiratory distress syndrome, neonatal pneumonia, necrotizing enterocolitis, and retinal disease, which cause a heavy burden to the family and society [3, 4]. sPTB is considered to be a multifactorial syndrome, related to aging, functional progesterone withdrawal, cervical disease, and stress [5]. However, its etiology and pathogenesis remain unclear, which attracts more attention in the latest years.

Many studies have mentioned that the pathogenesis of preterm birth is closely related to changes in the microenvironment of the maternal-fetal interface. Gomez-Lopez et al. and Boyle et al. have pointed out that it is prone to form an inflammation cascade, which leads to the occurrence of preterm birth when the inflammatory balance is disrupted at the maternal-fetal interface [6, 7]. We have known that the maternal-fetal interface is mainly composed of fetal membrane, decidual, and uterine. Among them, the fetal membrane is unique since it is not only functioning as an essential component separating the fetus from the mother's body, but also operating as a vital window for understanding the pathophysiology of preterm birth [8]. The fetal membrane is rich in the extracellular matrix (ECM). The degradation of ECM is often closely related to the incidence of preterm birth [9]. Furthermore, among all the different cells of the fetal membrane, amniotic epithelial cells have attracted the most attention since it is considered to be the earliest cell to receive fetal-derived signals [10]. The apoptosis of human amniotic epithelial cells is involved in preterm birth as well [11]. Meanwhile, human amniotic epithelial cells can be easily activated by external stimuli including lipopolysaccharide (LPS), hormone, and mechanical traction, and then subsequently releasing a large number of inflammatory factors, such as tumor necrosis factor-*α* (TNF-*α*), interleukin-1*β* (IL-1*β*), and interleukin-6 (IL-6), which ultimately mediate the excessive inflammation associated with preterm birth [12, 13].

Therefore, it is obvious to speculate that the most key link in excessive inflammation is the release of a large number of proinflammatory factors. In recent years, with the accumulation of data, it is found that interleukins play a critical role in regulating the release of proinflammatory factors. For instance, IL-33 could induce the expression of progesterone-induced blocking factor (PIBF1) by functioning the prevention effect of decidual B cells from preterm labor caused by LPS-induced proinflammatory factors [14]. Dambaeva et al. found that IL-22 can also prevent preterm birth and intrauterine fetal death by reducing the production of proinflammatory factors exposed to LPS [15]. As a typical representative of proinflammatory factors, IL-1 and its family members orchestrate numbers of inflammatory diseases, including preterm birth. However, interleukin-37 (IL-37) is a unique member of the IL-1 family, discovered in silico in 2000, which functions as a natural suppressor of inflammatory and immune responses. The gene of IL-37 is located at chromosome 2 and the molecular weight is about 17∼25 kDa.The structure of IL-37 is similar to the IL-1 family, which consists of twelve *β*-tube-like structures. It has six exons encoding five subtypes of IL-37, which are named as IL-37a, IL-37b, IL-37c, IL-37d, and IL-37e. In addition, IL-37 can be found in multiple human tissues, such as lung, thymus, bone marrow, and uterus tissues [16, 17]. Under physiological conditions, the expression of IL-37 is at a low level. However, once stimulated by inflammation, the expression of IL-37 will increase sharply [18]. Ellisdon et al. mentioned that IL-37 could significantly ameliorate LPS-induced inflammation in sepsis [19]. And Ye et al. indicated that IL-37 could inhibit the production of inflammatory cytokines in systemic lupus erythematosus [20]. Moreover, in the perinatal field, Southcombe and Yu et al. have demonstrated that IL-37 could be aberrantly expressed on the placenta and umbilical cord tissues in patients with preeclampsia and gestational diabetes mellitus, respectively [21, 22]. These studies mainly focus on the differences in expression. Little is known about the mechanisms. However, preterm birth is considered as a typical inflammatory disease, and no data have been found to reveal the role of IL-37. Therefore, we infer whether a deficiency of IL-37 in human fetal membranes is related to the pathogenesis of sPTB. This study is aimed at demonstrating that IL-37 can limit excessive inflammation and subsequently inhibit inflammation-induced ECM remodeling and apoptosis of human amniotic epithelial cells through the NF-*κ*B and IL-6/STAT3 pathway, which might further enrich theoretical strategies for sPTB.

## 2. Materials and Methods

### 2.1. Sample Collection

Subjects were enrolled from the First Affiliated Hospital of Chongqing Medical University and ethics approval was gained from the Ethics Committee of the First Affiliated Hospital of Chongqing Medical University. All the participants signed informed consent and recruited into the study if they were a singleton pregnancy. sPTB was defined according to the guidelines published by the *American Journal of Obstetrics and Gynecology*. All participants with chronic medical disorders were excluded, including diabetes mellitus, cardiovascular disease, infectious disease, autoimmune disease, chronic renal disease, chronic hypertension, and metabolic diseases. Fetal membrane tissues were collected immediately after delivery, and blood samples were taken from the vein from both patients with sPTB (*n* = 19) and control pregnant women (*n* = 21). Clinical information of study objects is shown in Table 1. After obtaining the samples, they were transferred to the laboratory with ice boxes within 1 hour and washed in sterile phosphate buffer solution for three times in order to remove the bloodstain on tissues. Blood samples were centrifuged at 3000 rpm for 10 minutes at 4°C. All the samples were stored at -80°C before analysis.

### 2.2. Cell Line and Cell Culture

The WISH (Wistar Institute Susan Hayflick) cell line was purchased from the commercialized company. Cells were cultured in MEM (Gibco, USA) complete medium containing 10% fetal bovine serum (PAN, Germany), 1% nonessential amino acid (Solarbio Biotechnology, Beijing, China), and 1% penicillin (110 U/ml) and streptomycin (100 *μ*g/ml) (Beyotime Biotechnology, Shanghai, China). Then, cells were incubated in 5% CO_2_ at 37°C.

### 2.3. Western Blot

Human amniotic epithelial cells treated with recombinant IL-37 protein (rhIL-37, R&D Systems, USA), recombinant IL-6 protein (rhIL-6, PeproTech, USA), and fetal membrane tissues were lysed in RIPA lysis buffer (MedChemExpress, USA). Total protein concentration was detected utilizing a BCA Protein Assay Kit (Beyotime Biotechnology, Shanghai, China). The antibodies against the following proteins were used: IL-37 (1 : 500; Santa Cruz, USA); Bax, Bcl-2, MMP9, T-NF-*κ*B, p-NF-*κ*B (Ser536), *β*-actin (1 : 1000; Cell Signaling Technology, USA); MMP2, STAT3, p-STAT3 (Tyr705) (1 : 1000; Affinity Biosciences, USA); *β*-tubulin, GAPDH (1 : 1000; Servicebio Biotechnology, Wuhan, China). The same volume of protein (40 *μ*g) was separated electrophoretically by SDS-PAGE and transferred to a PVDF membrane (Roche, Germany). After the transfer, the membranes were blocked with 5% skimmed milk for 1 hour and incubated overnight at 4°C with the indicated antibody. The next day, the membranes were incubated with an appropriate horseradish peroxidase-linked secondary antibody for 1 hour at room temperature. After incubation with the secondary antibody, the bands were visualized by an ECL. The secondary antibodies utilized in this research were goat anti-rabbit IgG-HRP (1 : 5000; Boster, Beijing, China) and goat anti-mouse IgG-HRP (1 : 5000; ZSGB-BIO, Beijing, China). Image Lab Software (Bio-Rad, California, USA) was used for imaging and ImageJ was used for densitometric analysis.

### 2.4. Quantitative RT-PCR

Total RNA was extracted from fetal membrane tissues and cells using TRIzol reagent (Thermo Fisher Scientific, USA) and quantified by measuring the ratio of A260nm/A280nm using NanoDrop (Thermo Fisher Scientific, USA). Total RNA was reverse transcribed using an EvoScript Universal cDNA Master Reagent Kit (Roche, Germany). The primers used in this study are listed in Table 2 (TSINGKE, Chongqing, China). Briefly, the real-time PCR reaction was consisted of 2 *μ*l of cDNA, 1 *μ*l of each primer, 5 *μ*l of SYBR Green qPCR Master Mix (MedChemExpress, USA), and 1 *μ*l of ultrapure water, for a total volume of 10 *μ*l, and was conducted in a Bio-Rad CFX-96 system (Bio-Rad, California, USA). The reaction conditions were as follows: 5 minutes at 95°C, 40 cycles of 10 seconds at 95°C, 20 seconds at 63.3°C, and 20 seconds at 72°C, respectively; melting curves were generated after each endpoint amplification for 10 seconds at 95°C, followed by 30-second increments of 0.5°C from 65 to 95°C. *β*-Actin was used as an endogenous reference. The 2^−△△Ct^ method was used for the calculation.

### 2.5. Immunofluorescence

When the bloodstain was removed from the tissue, the fetal membrane was fixed with 4% paraformaldehyde for 24 hours, and then dehydrated in 30% sucrose solution for 6 hours. Lastly, the tissues were embedded in optimal cutting temperature compound and sliced into sections. When conducting immunofluorescence, the slices firstly permeabilized with 0.1% Triton X-100 for 5 minutes and then blocked with 5% BSA for 1 hour at 37°C. The tissues were incubated overnight at 4°C with target antibodies at an appropriate dilution. The target antibodies IL-37 (1 : 50; Santa Cruz, USA) were used. The next day, tissues were dealt with the appropriate fluorescein-conjugated secondary antibodies against rabbit or mouse IgG (1 : 100; Abbkine Biotechnology, Wuhan, China). 6-Diamidino-2-phenylindole (DAPI, Servicebio Biotechnology, Wuhan, China) was utilized for nuclear staining.

### 2.6. Immunohistochemistry

Fetal membranes were fixed with 4% paraformaldehyde, embedded in paraffin, and sliced at a thickness of 3-5 *μ*m. The slices were dewaxed in xylene and dehydrated in gradient alcohol solutions. As to antigen retrieval, the slices were heated in sodium citrate buffer at high temperature for 4 minutes and middle temperature for 15 minutes in a microwave oven. Then, the endogenous peroxidase activity was removed with 3% H_2_O_2_ for 10 minutes and blocked with goat serum for 1 hour. Slices were incubated with IL-37 antibody (1 : 50, Santa Cruz, USA) at 4°C overnight. The next day, the sections were incubated with a biotin-labeled secondary antibody (ZSGB-BIO, Beijing, China). 3,3′-Diaminobenzidine (DAB) and hematoxylin (blue) were utilized to label the target and cell nuclei, respectively. Lastly, the stained sections were then evaluated by a Super Sensitive™ Link-Label IHC detection System (BioGenex, San Ramon, CA, USA).

### 2.7. Cell Apoptosis Assay

According to the manufacturer's protocols of the Annexin V-FITC kit (Beyotime Biotechnology, Beijing, China), the cells were plated into 6-well plates at 4 × 10^5^ cells/ml per well. Apoptosis was assessed by flow cytometry after the cells were transfected with IL-37 siRNA for 48 hours. The cells were collected and washed three times with cold phosphate buffer solution, and then mixed with the Annexin V-FITC and PI binding buffer for 20 minutes. Finally, the mixture was analyzed using a flow cytometer (BD Biosciences, San Jose, CA, USA).

### 2.8. EdU DNA Synthesis Assay

After transfected with IL-37 siRNA for 48 hours, cell proliferation assay was performed using a 5-ethynyl-20-deoxyuridine (EdU) detection kit (Ribobio, Guangzhou, China). According to the manufacturer's instructions, cells were dealt with 50 *μ*mol/l EdU for 2 hours at 37°C and then fixed with 4% paraformaldehyde for 30 minutes. After, cells were incubated with 2 mg/ml glycine for 5 minutes before treated with 0.5% Triton X-100 in phosphate buffer solution for 10 minutes and stained with 1× Apollo reaction cocktail for 30 minutes at room temperature. Subsequently, the counterstaining of cell nuclei was conducted with Hoechst. The proliferation rate was determined by the ratio of EdU staining-positive cells to total cells. Images were captured under a fluorescence microscope.

### 2.9. Cell Viability Assay

WISH cells were planted into 96-well plates (5000 cells/well) and then transfected with IL-37 siRNA for 48 hours after adhesion. Two fluorescent probes (Live/Death Cell Imaging Kit, Invitrogen, USA) (1 drop/500 *μ*l medium) were used in each well and then incubated for 15 minutes prior to photography under fluorescence microscopy. Six fields of view were captured for each group. Then, the counts of total and dead cells were calculated.

### 2.10. Transfection of siRNA

The sequences employed were IL-37 siRNA, 5′-UCUACUGUGACAAGGAUAATT-3′ and negative control siRNA (si_NC), 5′-UUCUCCGAACGUGUCACGUTT-3′. Those were designed and synthesized by GenePharma (Shanghai, China). Cells were planted into 6-well plates at a density of 10^5^ cells/well, and then transfected with IL-37 siRNA or negative control siRNA using Lipo 2000 reagent according to the recommended protocol. After transfection for 24 hours, cells were prepared for RT-qPCR and 48 hours for western blot. The transfection efficiency was confirmed by RT-qPCR and western blot.

### 2.11. ELISA Assay

The concentration of IL-37 in human peripheral blood plasma was measured by a human IL-37 ELISA kit (RayBiotech, USA). The ELISA kit used to measure the expression of IL-6 in cell supernatant was purchased from Elabscience (Wuhan, China), and the ELISA kits used to measure the expression of IL-1*β*, TNF-*α* in cell supernatant were purchased from 4A Biotech (Beijing, China). All the experiments were conducted according to the manufacturer's instructions. The absorbance of the supernatant was assessed at 450 nm in a MultiskanGO plate reader (Thermo Fisher Scientific, Waltham, MA, USA).

### 2.12. Gelatin Zymography

The total proteins of fetal membranes and cells were extracted using the same method as the western blot. The proteins were separated by 10% polyacrylamide gels containing sodium dodecyl sulfate (SDS) and 2 *μ*g/ml gelatin. After electrophoresis, the gels were washed with 2.5% Triton X-100 for 1.5 hours to remove the SDS and incubated for 48 hours with an incubating solution containing 50 mM Tri-HCl, 200 mM NaCl, 5 mM CaCl_2_, and 0.02% Brij-35 at 37°C. After incubation, the gels were stained in 0.5% *w*/*v* Coomassie brilliant blue R-250 at 37°C for 1 hour and then incubated with a decolorizing solution to visualize gelatinolytic bands. The different protease bands were qualitatively and quantitatively analyzed with Image Gel software (Bio-Rad, California, USA).

### 2.13. Statistical Analysis

Student's *t*-test was applied to determine the significant differences between the two groups. Comparison among multiple groups was performed by one-way ANOVA to detect the significant differences. All data were shown as the mean ± standard deviation (SD). All experiments were repeated at least three times. All statistical analysis was performed utilizing GraphPad Prism software, version 8.0 (GraphPad Software Inc., La Jolla, CA, USA). Results were considered statistically significant when *p* < 0.05 level.

## 3. Results

### 3.1. IL-37 Expression Significantly Decreases in sPTB Pregnancies

**Figure 1:**
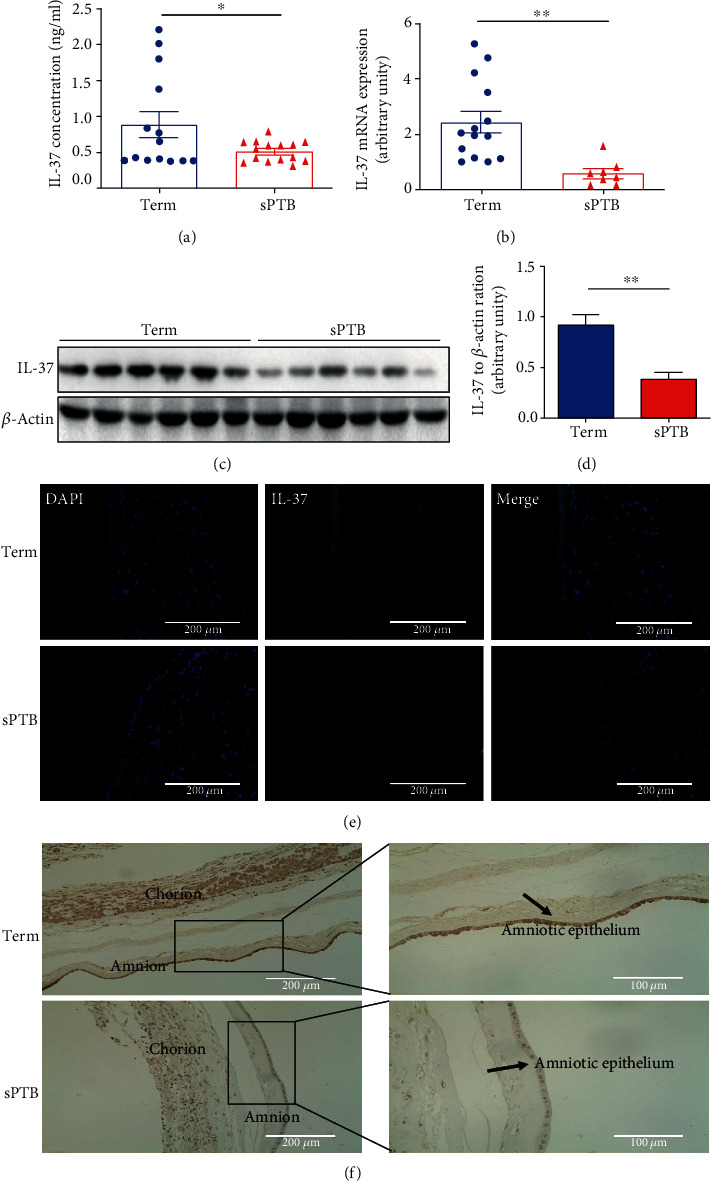
The expression and distribution of IL-37 in fetal membranes and plasma complicated with spontaneous preterm birth. (a) The plasmas from term (*n* = 14) and sPTB delivery women (*n* = 15) were collected, and IL-37 level was detected by ELISA. The detection range of IL-37 was 80-20000 pg/ml. (b) The mRNA expression level of IL-37 in fetal membranes was measured by quantitative real-time PCR analysis. The results were normalized to *β*-actin. (c) The fetal membranes from term (*n* = 6) and sPTB delivery women (*n* = 6) were harvested, and IL-37 level was detected by western blot. (d) Statistical analysis of western blot in the result (c). (e) Immunofluorescence staining was utilized to locate the expression site of IL-37 (green) in fetal membranes. Scale bar: 200 *μ*m. (f) Immunohistochemical staining of IL-37 in fetal membranes. Scale bar: 200 *μ*m and 100 *μ*m. ^∗^*p* < 0.05; ^∗∗^*p* < 0.01.

Firstly, we compared the expression of IL-37 in the preterm and the term groups. The results showed that IL-37 was significantly decreased in the peripheral blood plasma of sPTB compared to term labor [0.89 ± 0.18 vs. 0.52 ± 0.04, *t* = 2.094, *p* = 0.046; (Figure 1(a))]. In addition, the mRNA and protein expression levels of IL-37 were also lower in fetal membranes of the sPTB group than those of the term group [2.46 ± 0.38 vs. 0.61 ± 0.16, *t* = 3.520, *p* = 0.0022; Figure 1(b); 0.92 ± 0.10 vs. 0.39 ± 0.06, *t* = 4.419, *p* = 0.0013; Figures 1(c) and 1(d)]. Moreover, immunofluorescence (IF) and immunohistochemistry (IHC) were utilized. As shown in Figures 1(e) and 1(f), IL-37 was localized at human amnion and chorion layers. Collectively, these results indicated that the expression of IL-37 in human peripheral plasma and fetal membranes was significantly lower in the sPTB group than that in the term group.

### 3.2. IL-37 Limits Excessive Inflammation

**Figure 2:**
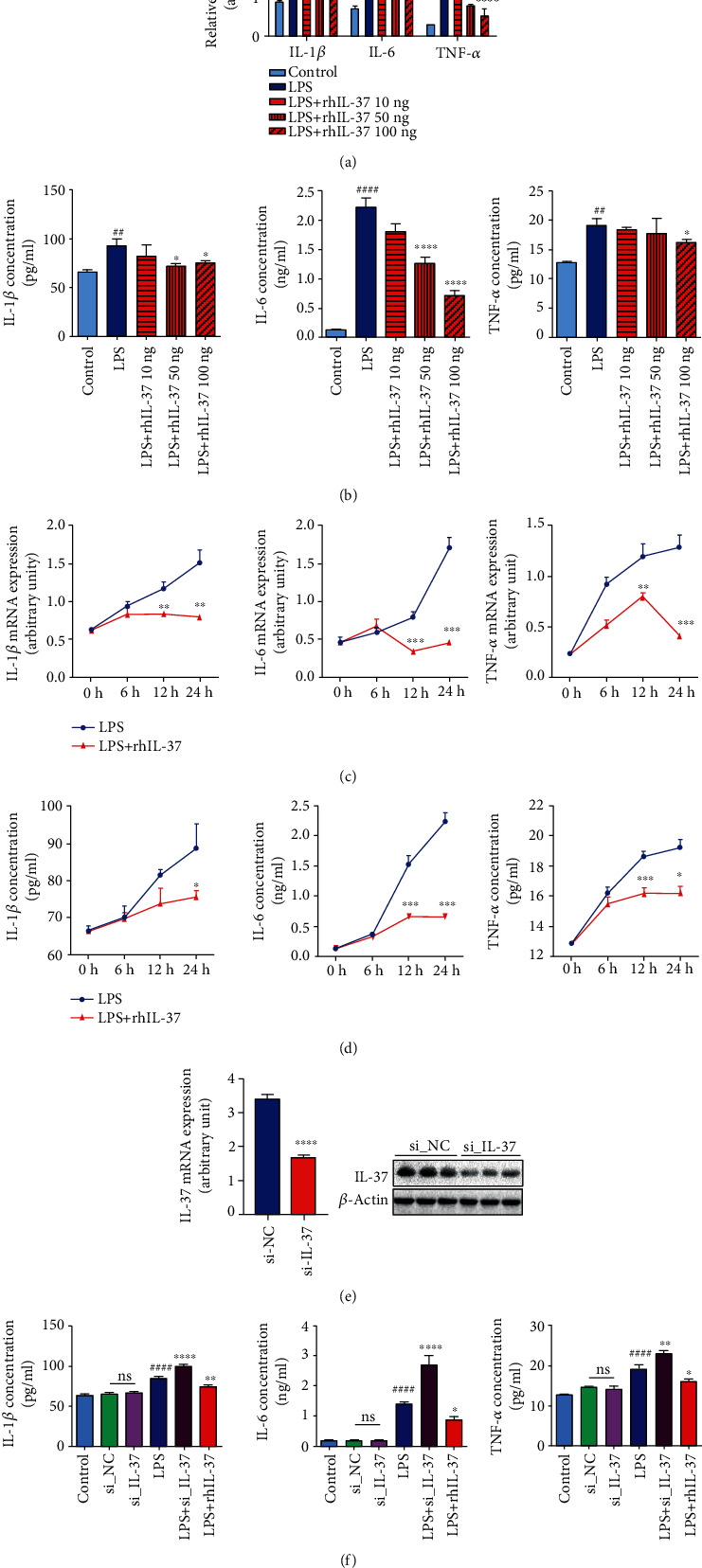
IL-37 suppressed expression of inflammatory factors in human amniotic epithelial cells. (a, b) Dose-dependent effects of IL-37 (10 ng/ml, 50 ng/ml, and 100 ng/ml) on the mRNA and protein expression levels of IL-1*β*, IL-6, and TNF-*α* were measured by quantitative real-time PCR and ELISA. The detection range of the ELISA kit was 7.8-1000 pg/ml. The results were normalized to *β*-actin. (c, d) Time-dependent effects of 100 ng/ml IL-37 (0 h, 6 h, 12 h, 24 h) on the mRNA and protein expression levels of IL-1*β*, IL-6, and TNF-*α* were detected by quantitative real-time PCR and ELISA. The detection range of the ELISA kit was 7.8-1000 pg/ml. The results were normalized to *β*-actin. (e) Transfection efficiency of IL-37 siRNA was detected by quantitative real-time PCR and western blot. The results were normalized to *β*-actin. (f) The concentrations of IL-1*β*, IL-6, and TNF-*α* were measured by ELISA in the cell supernatant of the LPS-treated WISH cells after transfection with IL-37 siRNA for 48 hours and treatment with LPS and rhIL-37 for 24 hours, respectively. The detection range of the ELISA kit was 7.8-1000 pg/ml. ns: nonsignificance; ^∗^*p* < 0.05 vs. LPS; ^∗∗^*p* < 0.01 vs. LPS; ^∗∗∗^*p* < 0.001 vs. LPS; ^∗∗∗∗^*p* < 0.0001; ^##^*p* < 0.01 vs. control; ^####^*p* < 0.0001.

After we determined that IL-37 did significantly decrease in fetal membranes of the sPTB group, we are wondering how it works. To determine the effect of IL-37 on cytokine production in WISH cells, quantitative real-time PCR and ELISA were used to screen various inflammatory cytokines. When WISH cells were treated with both LPS (1 *μ*g/ml) and rhIL-37 (10 ng/ml, 50 ng/ml, 100 ng/ml) for 6 hours, 12 hours, and 24 hours, the mRNA and protein expressions of IL-1*β*, IL-6, and TNF-*α* decreased in a dose- and time-dependent manner (Figures 2(a)–2(d)). The most significant decrease occurred at a concentration of 100 ng/ml and time point 24 hours, so this concentration and time point were selected for further studies. Next, we transfected IL-37 siRNA (si_IL-37) and negative control siRNA (si_NC) into WISH cells, and their transfection efficiencies were confirmed by quantitative real-time PCR and western blot (Figure 2(e)). Then, experiments were carried out. The results showed that knockdown of IL-37 significantly increased the mRNA and protein expressions of downstream inflammatory factors (IL-1*β*, IL-6, and TNF-*α*) when exposed to LPS, which were reversed by the administration of rhIL-37 (Figure 2(f)). However, the IL-37 siRNA group no longer downregulated the expression of IL-1*β*, IL-6, and TNF-*α* in the absence of LPS stimulation. Dramatically, among the three inflammatory factors, the change of IL-6 was the most obvious one.

### 3.3. IL-37 Inhibits ECM Degradation

**Figure 3:**
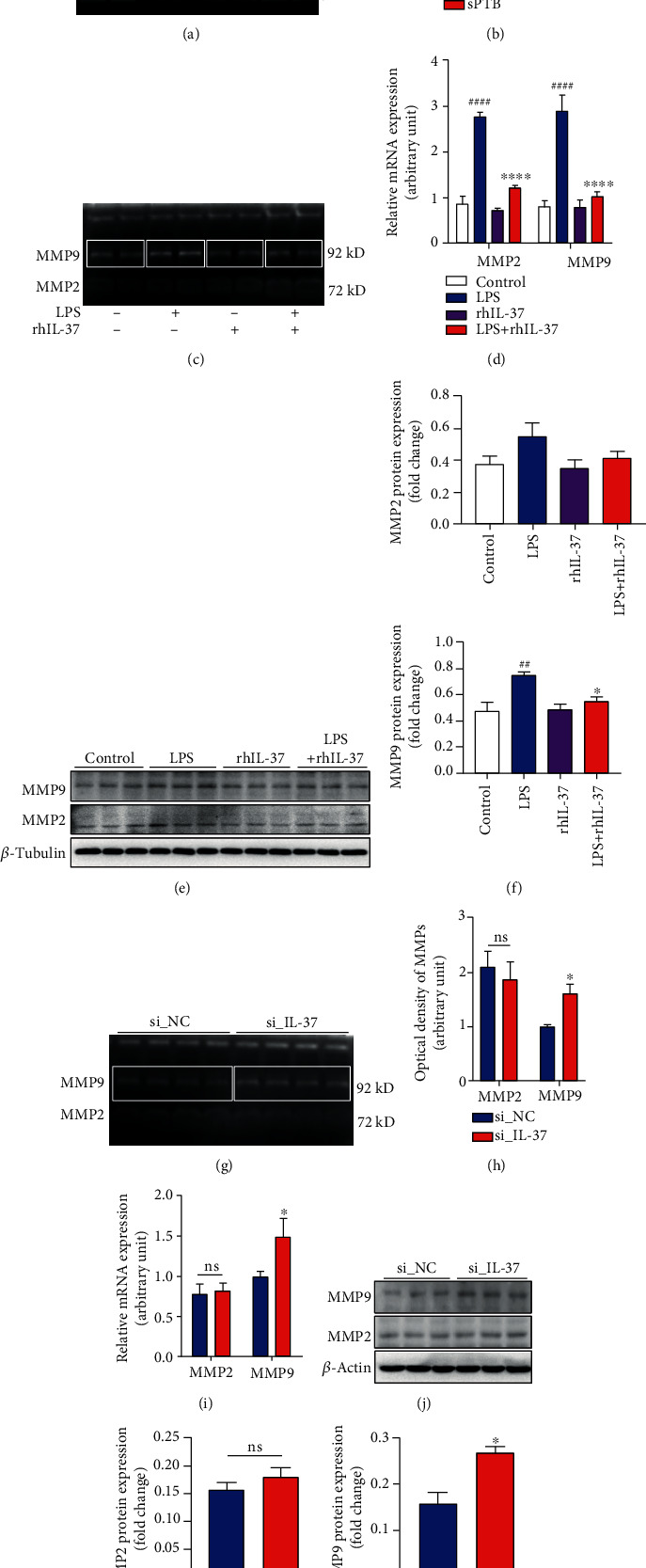
Effects of IL-37 on expression and activity of MMP2 and MMP9 in fetal membranes and human amniotic epithelial cells. (a) The activities of MMP2 and MMP9 in fetal membranes collected from term (*n* = 4) and sPTB (*n* = 4) delivery women were measured with gelatin zymogram. (b) Statistical analysis of gelatin zymogram in the result (a). (c) The activity of MMP2 and MMP9 in WISH cells following treatment with LPS and rhIL-37 for 24 hours was detected with gelatin zymogram. (d) The mRNA expression levels of MMP2 and MMP9 after exposed to LPS and rhIL-37 for 24 hours were detected by quantitative real-time PCR analysis. The results were normalized to *β*-actin. (e) The protein expression levels of MMP2 and MMP9 after exposed to LPS and rhIL-37 for 24 hours were measured by western blot. (f) Statistical analysis of western blot in the result (e). (g) The activity of MMP2 and MMP9 between the IL-37 siRNA group and the si_NC group was detected with gelatin zymogram. (h) Statistical analysis of gelatin zymogram in results (g). (i) The mRNA expression levels of MMP2 and MMP9 were measured by quantitative real-time PCR analysis in each group. The results were normalized to *β*-actin. (j) The protein expression levels of MMP2 and MMP9 between the IL-37 siRNA group and the si_NC group were detected by western blot. (k) Statistical analysis of western blot in the result (j). ns: nonsignificance; ^∗^*p* < 0.05 vs. LPS; ^∗∗∗∗^*p* < 0.0001; ^##^*p* < 0.01 vs. control; ^####^*p* < 0.0001.

The results showed that IL-37 can inhibit the release of downstream inflammatory factors in fetal membranes and thus limit excessive inflammation. Previous studies have revealed that IL-37 can inhibit the release of matrix metalloproteinases (MMPs) in other diseases, such as endometriosis [23]. Therefore, we speculated that IL-37 will regulate the effect of MMPs in preterm birth. Firstly, the role of MMP2 and MMP9 was verified (Figure 3(a)). As presented in Figure 3(b), the activity of MMP9 was notably elevated in fetal membranes of the preterm birth group. Then, the effect of rhIL-37 on MMP2 and MMP9 activity and expression in WISH cells were measured. Our data indicated that rhIL-37 treatment significantly reduced the mRNA expressions of MMP2 and MMP9 induced by LPS (Figure 3(d)). However, similar results were only observed in the protein level of MMP9 (Figures 3(e) and 3(f)). Although MMP2 was also decreased, the difference was not statistically significant. In addition, rhIL-37 significantly reduced the LPS-induced activity of MMP9, whereas MMP2 was not affected (Figure 3(c)). Correspondingly, the siRNA-mediated knockdown of IL-37 significantly upregulated the activity of MMP9, whereas had no effect on MMP2 (Figures 3(g) and 3(h)), which was in accordance with the results found in mRNA (Figure 3(i)) and protein levels (Figures 3(j) and 3(k)). However, the administration of rhIL-37 alone produced no effect on the expression and activity of MMP2 and MMP9 (Figures 3(c)–3(f)).

### 3.4. IL-37 Inhibits Apoptosis of Human Amniotic Epithelial Cells

**Figure 4:**
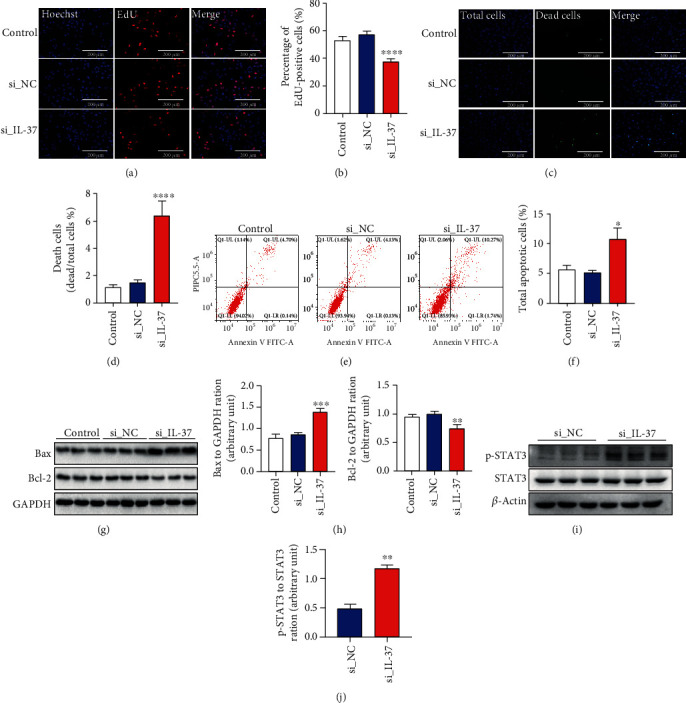
Effects of IL-37 on apoptosis and proliferation in human amniotic epithelial cells. (a) Cell proliferation between the IL-37 siRNA group and the si_NC group was measured by 5-ethynyl-20-deoxyuridine (EdU) incorporation assay. The blue color indicates the nuclei, and the red color represents the EdU-positive nuclei. Scale bar: 200 *μ*m. (b) Statistical analysis of EdU staining in the result (a). (c) The Live/Death cell kit was used to analyze the number of dead cells between the IL-37 siRNA group and the si_NC group. The blue color indicates the nuclei, and the green color represents the death cells. Scale bar: 200 *μ*m. (d) Statistical analysis of death cell rate in the result (c). (e) Cell apoptosis between the IL-37 siRNA group and the si_NC group was assessed by the flow cytometer. Representative pictures of flow cytometry for apoptosis rate. (f) Statistical analysis of flow cytometry in the result (e). (g) The apoptosis-related protein biomarkers were measured by western blot in each group. (h) Statistical analysis of western blot in the result (g). (i) The STAT3 signaling was measured by western blot between the si_NC group and the IL-37 siRNA group. (j) Statistical analysis of western blot in the result (i). ∗*p* < 0.05 vs. si_NC; ^∗∗^*p* < 0.01; ^∗∗∗^*p* < 0.001; ^∗∗∗∗^*p* < 0.0001.

Cell proliferation and apoptosis maintain life activities. However, the onset of preterm birth is inseparable from the proliferation and apoptosis of amniotic epithelial cells [9]. Therefore, the effect of IL-37 on the proliferation and apoptosis of amniotic epithelial cells was investigated. EdU staining showed that knockdown of IL-37 significantly reduced DNA synthesis in WISH cells [51.43% ± 0.52 vs. 32.54% ± 0.34, *p* < 0.0001; Figures 4(a) and 4(b)]. Furthermore, the ratio of dead cells to total cells was observably increased in the IL-37 siRNA group compared with the si_NC group [1.71% ± 0.21 vs. 4.69% ± 0.84, *p* < 0.0001; Figures 4(c) and 4(d)]. Hence, it appears that IL-37 deficiency can not only inhibit proliferation but also promote cell death. In line with these data, the percentage of apoptotic cells was detected remarkably augmented with flow cytometry after the transfection of IL-37 siRNA into WISH cells [5.11% ± 0.61 vs. 10.70% ± 3.26, *p* = 0.0276; Figures 4(e) and 4(f)]. In order to identify the influence of IL-37 on apoptosis-related proteins, the expression of Bcl-2 and Bax was analyzed by western blot (Figure 4(g)). As shown in Figure 4(h), the expression of Bax was significantly upregulated in the IL-37 siRNA group when contrast to the si_NC group, while Bcl-2 decreased. Additionally, compared with the si_NC group, phosphorylated STAT3 (p-STAT3) was activated in the IL-37 siRNA group (Figures 4(i) and 4(j)). These data indicated that IL-37 could efficiently inhibit apoptosis by regulating antiapoptotic molecule Bcl-2 and proapoptotic molecule Bax via the STAT3 pathway.

### 3.5. IL-37 Exerts Anti-Inflammatory Effects via the NF-*κ*B and IL-6/STAT3 Signaling Pathway

**Figure 5:**
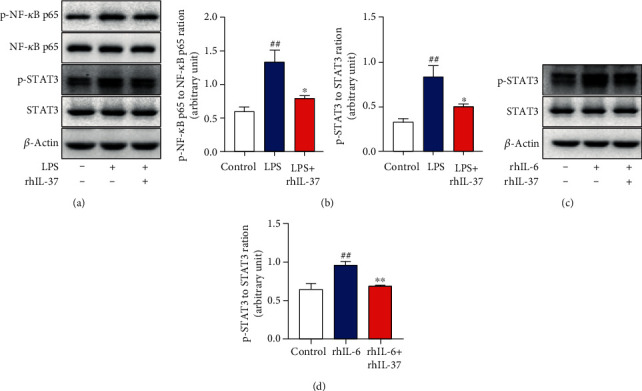
IL-37 exerts its biological functions through the NF-*κ*B and IL-6/STAT3 signaling pathway. (a) The phosphorylation of NF-*κ*B, STAT3, and total amounts of NF-*κ*B, STAT3 were detected by western blot after administrated by LPS and LPS+rhIL-37 for 12 hours. (b) Densitometry quantifications of p-NF-*κ*B to T-NF-*κ*B and p-STAT3 to T-STAT3 level were shown. (c) Western blot analysis of p-STAT3 and T-STAT3 expression was presented following treatment with rhIL-6 and rhIL-37+rhIL-6 for 12 hours. (d) Densitometry quantification of p-STAT3 to T-STAT3 level was demonstrated. ^∗^*p* < 0.05 vs. LPS; ^∗∗^*p* < 0.01 vs. rhIL-6; ^##^*p* < 0.01 vs. control.

To further explore the molecular mechanism of IL-37 on the process of inflammation induced by LPS, the effects of IL-37 on the NF-*κ*B and STAT3 signaling in WISH cells were examined (Figure 5(a)). The western blot data showed that rhIL-37 obviously attenuated phosphorylation of the NF-*κ*B and STAT3 induced by LPS in WISH cells (Figure 5(b)). Meanwhile, the WISH cells were administrated by rhIL-6 and rhIL-6+rhIL-37 to determine whether the IL-6/STAT3 signaling pathway was involved in the IL-37-mediated anti-inflammatory effects. As shown in Figures 5(c) and 5(d), phosphorylation of STAT3 was activated after treated with rhIL-6, while reversed by rhIL-37. These results indicated that IL-37 might exert its anti-inflammatory effects mainly through the NF-*κ*B and IL-6/STAT3 signaling pathway in human amniotic epithelial cells.

## 4. Discussion

sPTB is a leading cause of neonatal mortality and morbidity for children under 5 years old worldwide, which carries a heavy economic burden on families and society [24]. It is a multifactorial syndrome, and researches on the mechanism of inflammation have received widespread attention in the latest years. The inflammatory factors such as IL-1*β*, TNF-*α*, and IL-6 were all reported to involve in the pathogenesis of preterm birth [25, 26]. Elevated concentration of TNF-*α* in the amniotic fluid activated nuclear factor *κ*B (NF-*κ*B) pathway, triggering amplification of inflammatory processes that lead to preterm birth [27, 28]. Recently, increased IL-6 levels in amniotic fluid from the second trimester were associated with the onset of sPTB, which makes it a potential biomarker [29, 30]. As we know, IL-1*β*, as the most intensively researched molecule in the field of preterm birth, has always been considered as the molecule that dominates the inflammatory cascade at the maternal-fetal interface [31]. The IL-1 family is made up of 11 different cytokines, which present the same structure and share a similar function. However, the pathways through which they are activated are quite different from one to another. To our surprise, the members of the IL-1 family can directly interact with each other. Gallenga et al. and Franza et al. have indicated that IL-37 could inhibit IL-1-mediating activities and IL-38 could inhibit the proinflammatory activity of IL-36 [32, 33]. As a member of the IL-1 family, IL-37 exerts a completely different anti-inflammatory effect from IL-1*β*. Nold et al. found that IL-37 releasing from epithelium and macrophages almost completely suppressed the expression of several proinflammatory factors (mainly IL-1*β*, IL-6, and TNF-*α*) *in vitro*, and protected mice against sepsis induced by LPS *in vivo* [34]. It has been reported that IL-37 performs a protective effect on colitis, arthritis, pancreatitis, and other inflammatory diseases which were induced by exogenous stimuli [16]. However, the role of IL-37 in preterm birth, also an inflammatory disease, has not been clearly reported yet. Therefore, we hypothesized that IL-37 will affect the occurrence of preterm birth by limiting the excessive inflammatory effect at the maternal-fetal interface.

Firstly, we are wondering whether IL-37 has different expressions in maternal tissues between the preterm birth group and the control group. The results showed that the expression of IL-37 in the plasma and fetal membranes was decreased when compared with that in the term group, which suggested that IL-37 might be associated with the occurrence of preterm birth. Next, we verify whether IL-37 really exerts anti-inflammatory effects at the cellular level with the human amniotic epithelial cell line (WISH). Our data showed that IL-37 indeed suppressed the expression of inflammatory factors (IL-1*β*, IL-6, and TNF-*α*). As we know, the inflammatory cascade, mediating by different inflammatory factors, such as IL-1*β*, IL-6, and TNF-*α*, plays a crucial role at the maternal-fetal interface, which involves in uterine contractility and membrane rupture related to sPTB. Notably, among the three above inflammatory factors, the level of IL-6 showed the most significant change in WISH cells. Previous studies have shown that the resident cells of the chorion and decidua are capable of synthesizing and secreting IL-6, and its production is stimulated by LPS or proinflammatory factors such as IL-1*β* and TNF-*α* [35]. Furthermore, another study has demonstrated that IL-6 secretion is upregulated in fetal membranes of preterm pregnancies, indicating that IL-6 might be a key factor in accelerating the events of preterm birth [36]. Therefore, it is understandable that the change of IL-6 is the most obvious one among these markers. In summary, IL-37 is capable of inhibiting the production of IL-1*β*, IL-6, and TNF-*α*, which may play a vital role in the field of premature treatment.

The fetal membrane is not only an important mechanical barrier between the mother and the fetus but also an essential front line for inflammatory reactions. Rupture of the fetal membrane may cause a positive feedback loop for inflammation leading to preterm birth [9]. However, the strength and flexibility of the fetal membrane are primarily dominated by the key molecules of ECM remodeling, MMP2 and MMP9 [37]. A series of studies have identified that MMP2 and MMP9 could cause the reticulin proteolysis that results in ECM remodeling, which promotes membrane rupture that lastly leads to preterm birth [8]. It has been shown that IL-1*β*, TNF-*α*, and IL-6 can induce the expression of MMP2 and MMP9 in amniotic fluid and fetal membranes [38, 39]. According to our results, IL-37 could inhibit the production of IL-1*β*, TNF-*α*, and IL-6 in human amniotic epithelial cells, so we speculate that IL-37 will inhibit the MMP2 and MMP9 expressions. To clarify this hypothesis, the expressions of MMP2 and MMP9 in WISH cells were measured after treatment with IL-37. Surprisingly, the MMP9 expression was found significantly downregulated after IL-37 treatment. However, no differences were found in MMP2 expression. According to the previous studies, IL-37 indeed affects MMP2 and MMP9 expressions in endometriosis, atherosclerosis, and human lung adenocarcinoma [40–42]. But why changes only occur in MMP9. In fact, amniotic epithelial cells exclusively produce MMP9, while amniotic mesenchymal cells produce only MMP2. It is therefore concluded that MMP2 and MMP9 exhibited cell-specific expression in the human fetal membranes [43]. Therefore, it is understandable that IL-37 can only regulate MMP9 instead of MMP2 in amniotic epithelial cells.

On the other hand, preterm birth is mediated by cell apoptosis, which may be affected by factors such as infections and external stimuli [9]. The risk signals stimulate Toll-like receptors which lead to the activation of NF-*κ*B and subsequently increase expression of the major proinflammatory factors including IL-1*β*, IL-6, and TNF-*α* in fetal membranes [11]. Among them, TNF-*α* could band to its receptor that initiates signal transduction through the TNFR-associated death domain (TRADD), which activates caspase activity eventually resulting in apoptosis [28]. IL-1*β* and IL-6 could increase the fragmentation of nuclei leading to apoptosis as well [44]. It is already verified that IL-37 could limit excessive inflammation in this study and several published articles have revealed that IL-37 could influence apoptosis in cervical cancer and renal carcinoma [45, 46]. Therefore, we want to investigate whether IL-37 can inhibit the apoptosis of amniotic epithelial cells in preterm birth. As is known to all, Bax and Bcl-2 are homologous proteins in the mitochondrial apoptosis pathway. The imbalance of Bax/Bcl-2 is capable of leading to apoptosis. Fortunato et al. have reported that the expression of Bax was increased, while Bcl-2 was decreased in the fetal membranes of the sPTB pregnancies [47]. A recent study suggested that IL-37 could inhibit Bcl-2 through STAT3 signaling [48]. Hence, we speculate that IL-37 is also able to suppress apoptosis by affecting the balance of Bax/Bcl-2 via the STAT3 pathway. According to our study, the results showed that the downregulation of IL-37 could promote apoptosis by increasing the expression of proapoptotic protein Bax and decreasing antiapoptotic protein Bcl-2 in WISH cells. The consistent results were shown in flow cytometry analysis. Furthermore, as shown in Figure 4(i) and Supplementary Figure 1, both knockdown and treatment with IL-37 both impacted STAT3 signaling. Thus, it is proved that IL-37 can affect preterm birth by regulating apoptosis through the STAT3 signaling pathway.

According to the above results, IL-37-mediated anti-inflammatory effects play a critical role in sPTB. Next, we tentatively explore its specific molecular mechanism. LPS, the component of Gram-negative bacteria, can generally activate the NF-*κ*B signaling pathway, which provokes a wide variety of inflammation responses and triggers the production of proinflammatory cytokines, including IL-1*β*, IL-6, and TNF-*α* [49]. According to our results (Figure 5(a)), the NF-*κ*B signaling pathway was activated after stimulated by LPS. Meanwhile, as shown in Figure 2(f), IL-1*β*, IL-6, and TNF-*α* were all increased, particularly IL-6. Previous studies have indicated that IL-6, the most well-known traditional activator of STAT3, was a hallmark of many human chronic inflammatory states, including sepsis, rheumatoid arthritis (RA), and inflammatory bowel disease (IBD) [50]. Hence, it could conceivably be hypothesized that LPS can exert its effects through the IL-6/STAT3 signaling pathway indirectly in sPTB. Basing on our results, STAT3 was upregulated after treated with IL-6. Therefore, it is understandable that LPS could activate phosphorylation of the STAT3 pathway through IL-6 indirectly. Previous studies suggested that IL-37 could inhibit the activation of the NF-*κ*B and IL-6/STAT3 signaling pathway and suppressed the occurrence of the inflammatory response in Myasthenia Gravis (MG) by affecting Tfh and B cells, and in lung cancer by affecting A549 cells [46, 51]. However, the anti-inflammatory effect of IL-37 via the NF-*κ*B and IL-6/STAT3 pathway in preterm birth remains elusive. The results showed that the expression of p-NF-*κ*B and p-STAT3 was significantly increased in the LPS-treated group, whereas significantly decreased after incubation with IL-37. Thus, it can be suggested that LPS activates the NF-*κ*B signaling pathway and then to trigger the production of proinflammatory factors (mainly IL-6), which subsequently phosphorylates STAT3. However, the above effects are reversed by IL-37.This study was confirmed by a previous study, further stating that IL-37 harnesses the anti-inflammatory properties of the signaling molecule NF-*κ*B and STAT3 [52].

Although we have conducted in vitro studies to verify our hypothesis, there are still some details that need further refinement. For the consistency of the results, the amniotic epithelial cells applied in our *in vitro* study are a cell line instead of the primary amniotic epithelial cells. Thus, we plan to address some of these issues in the next experiment, and *in vivo* study is also being scheduled. Furthermore, Masterson et al. have indicated that mesenchymal stem cells (MSCs) and their derivatives are incapable of modulating IL-37, and MSCs expressing IL-37 may have an enhanced therapeutic efficacy [53]. Actually, the fetal membrane, especially amnion, is full of MSCs, which may produce efficient IL-37 to maintain the immune balance at the maternal-fetal interface. Hence, IL-37 may be a therapeutic target for sPTB in the near future. However, more researches are needed.

## 5. Conclusion

In summary, the anti-inflammatory effect of IL-37 in fetal membranes by suppressing the NF-*κ*B and IL-6/STAT3 signaling pathway can inhibit apoptosis and remodeling of ECM and participate in preterm birth. These findings may further enrich the theoretical strategies for preterm birth.

## Figures and Tables

**Table 1:** Clinical information of study objects.

Maternal characteristics	Normal pregnancy (*n* = 21)	Spontaneous preterm birth (*n* = 19)
Maternal age (years)	27.86 ± 2.67	28.16 ± 3.18
Gestational age (weeks)	39.78 ± 0.78	33.40 ± 3.30^b^
Prepregnancy BMI (kg/m^2^)	20.80 ± 2.30	21.17 ± 2.36
Neonatal birth weight (g)	3351 ± 395.80	2337 ± 525.90^b^
fFN (positive or negative)	Negative	Negative
Placental weight (g)	586.20 ± 104.30	496.30 ± 85.26^a^

BMI: body mass index. Data are presented as the mean ± SD. ^a^*p* < 0.01; ^b^*p* < 0.0001.

**Table 2:** Characteristic of primers.

Genes	Sense primer (5′ → 3′)	Antisense primer (5′ → 3′)
IL-1*β*	CCACAGACCTTCCAGGAGAAT	GTGCACATAAGCCTCGTTATCC
IL-6	CCTAGAGTACCTCCAGAACAGA	CAGGAACTGGATCAGGACTTT
TNF-*α*	ACCTCTCTCTAATCAGCCCTCT	GGGTTTGCTACAACATGGGCTA
IL-37	TTCTTTGCATTAGCCTCATCCTT	CGTGCTGATTCCTTTTGGGC
MMP2	AAGGACAGCCCTGCAAGTTT	GTTCCCACCAACAGTGGACA
MMP9	GGTGATTGACGACGCCTTTG	GGACCACAACTCGTCATCGT
*β*-Actin	TGGCACCCAGCACAATGAA	CTAAGTCATAGTCCGCCTAGAAGCA

## Data Availability

The data used to support the findings of this study are available from the corresponding author upon request.

## Supplementary Materials

Supplementary Materials Figure S1: effects of IL-37 on apoptosis through IL-6 in human amniotic epithelial cells. (a) The expression of Bax and Bcl-2 was detected by western blot after treatment with rhIL-6 and rhIL-6+rhIL-37 for 12 hours. (b) Statistical analysis of western blot in the result (a). ^∗∗^*p* < 0.01 vs. rhIL-6; ^#^*p* < 0.05 vs. control; ^###^*p* < 0.001.
