# Supplementary material for: IL-37 Exerts Anti-Inflammatory Effects in Fetal Membranes of Spontaneous Preterm Birth via the NF-κB and IL-6/STAT3 Signaling Pathway
Source: Mediators Inflamm. 2020 Jul 11;2020:1069563. doi: 10.1155/2020/1069563 (PMC7369678; doi:10.1155/2020/1069563)
Supplement: Supplementary Materials — Figure S1: effects of IL-37 on apoptosis through IL-6 in human amniotic epithelial cells. (a) The expression of Bax and Bcl-2 was detected by western blot after treatment with rhIL-6 and rhIL-6+rhIL-37 for 12 hours. (b) Statistical analysis of western blot in the result (a). ∗∗p < 0.01 vs. rhIL-6; #p < 0.05 vs. control; ###p < 0.001. [file 1069563.f1.docx]

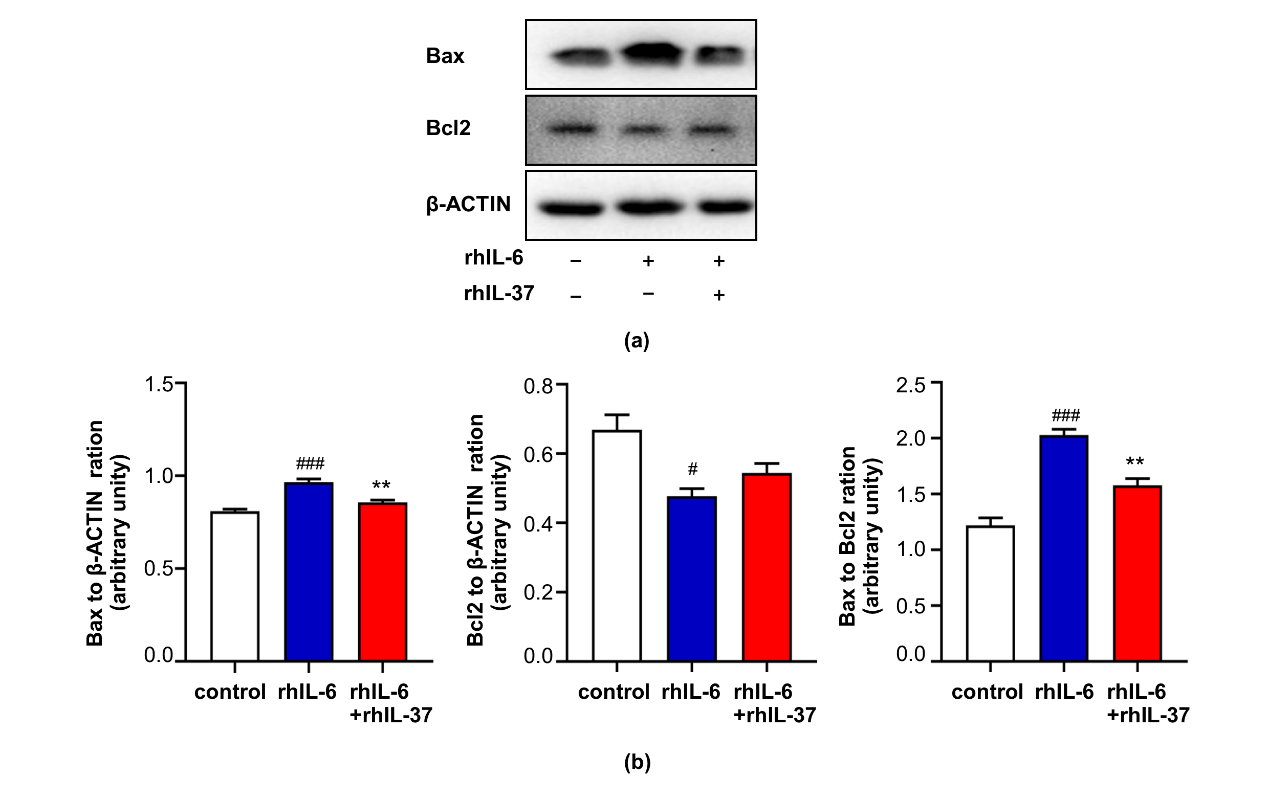


**Figure S1.** *Effects of IL-37 on apoptosis through IL-6 in human amniotic epithelial cells.* (a) The expression of Bax and Bcl-2 was detected by western blot after treatment with rhIL-6 and rhIL-6+rhIL-37 for 12 hours. (b) Statistical analysis of western blot in the result (a). **p<0.01vs. rhIL-6; #p<0.05 vs. control; ###p<0.001.
